# Genome-Wide Identification of Soybean ABC Transporters Relate to Aluminum Toxicity

**DOI:** 10.3390/ijms22126556

**Published:** 2021-06-18

**Authors:** Junjun Huang, Xiaoyu Li, Xin Chen, Yaru Guo, Weihong Liang, Huahua Wang

**Affiliations:** College of Life Science, Henan Normal University, Xinxiang 453007, China; jjhuang2012@163.com (J.H.); 40064007@163.com (X.L.); 36229237@163.com (X.C.); 041168@htu.edu.cn (Y.G.); whliang68@163.com (W.L.)

**Keywords:** ABC transporter proteins, soybean, molecular evolution, expression analysis, genomic survey, aluminum toxicity

## Abstract

ATP-binding cassette (ABC) transporter proteins are a gene super-family in plants and play vital roles in growth, development, and response to abiotic and biotic stresses. The ABC transporters have been identified in crop plants such as rice and buckwheat, but little is known about them in soybean. Soybean is an important oil crop and is one of the five major crops in the world. In this study, 255 *ABC* genes that putatively encode ABC transporters were identified from soybean through bioinformatics and then categorized into eight subfamilies, including 7 *ABCAs*, 52 *ABCBs*, 48 *ABCCs*, 5 *ABCDs*, 1 *ABCEs*, 10 *ABCFs*, 111 *ABCGs*, and 21 *ABCIs*. Their phylogenetic relationships, gene structure, and gene expression profiles were characterized. Segmental duplication was the main reason for the expansion of the *GmABC* genes. Ka/Ks analysis suggested that intense purifying selection was accompanied by the evolution of GmABC genes. The genome-wide collinearity of soybean with other species showed that GmABCs were relatively conserved and that collinear ABCs between species may have originated from the same ancestor. Gene expression analysis of *GmABCs* revealed the distinct expression pattern in different tissues and diverse developmental stages. The candidate genes *GmABCB23*, *GmABCB25*, *GmABCB48*, *GmABCB52*, *GmABCI1*, *GmABCI5*, and *GmABCI13* were responsive to Al toxicity. This work on the *GmABC* gene family provides useful information for future studies on ABC transporters in soybean and potential targets for the cultivation of new germplasm resources of aluminum-tolerant soybean.

## 1. Introduction

ATP-binding cassette (ABC) transporters are an ancient and huge transmembrane protein family that widely exist in natural organisms, and they have attracted extensive attention due to their diverse biological functions. ABC proteins release energy mainly by binding and hydrolyzing ATP, leading to conformational changes of proteins, thus realizing the transmembrane transport of a wide array of substrates, including carbohydrates, lipids, peptides, terpenes, cell metabolites, heavy metal chelates, and metal ions [1].

ABC transporters generally contain two domains: hydrophobic transmembrane domains (TMDs) containing four to six transmembrane helices and nucleotide-binding domains (NBDs) containing several highly conserved characteristic motifs, such as Walker A motif [GXXGXGKS/T], Walker B motif [(RK)X_3_GX_3_L(hydrophobic)_3_], ABC signature motif, the Q-loop, and the H-loop [2]. TMDs serve as recognition agents and channels for substrate transport lipid bilayers, whereas NBDs provide energy for substrate transport or non-transport processes through ATP binding and ATP hydrolysis.

On the basis of the phylogenetic analysis of ABC transporters, the similarity of conserved sequences, and the organization of their domains, ABC transporters are divided into eight subfamilies: ABCA—ABCG and ABCI. To date, the number of ABC transporter genes identified in plants is much higher than that of animals and microorganisms [3]. *Arabidopsis thaliana*, *Oryza sativa* [4], *Brassica campestris* [5], and *Lycopersicon esculentum* [6] have 129, 121, 314, and 154 putative ABC transporters, respectively. Plants have so many ABC transporters, and a diversity of substrates that promote ABC transporters are involved in many processes of plant life activities, such as regulating the transport of plant hormones, enriching and excreting toxic substances, secreting secondary metabolites, maintaining cell osmotic homeostasis, resisting various stresses, ingesting nutrients, transducing signals, and resistance response. In particular, with the increasingly prominent environmental problems, ABC transporters have attracted wide attention as they play roles in the detoxification of toxic compounds or heavy metals. For instance, AtABCB25 confers heavy metal tolerance, probably as glutathione disulfide transporters [7]. HvMDR2, a homolog of AtABCB27, is responsible for Fe sequestration [8]. CjMDR1 transports berberine [9]. AtABCC1 and AtABCC2 are related to cadmium and mercury by vacuolar sequestration [10]. AtABCC3, AtABCC6, AtABCC7, AtABCG32, CrCds1, OsABCG31, and OsABCG36 mediate tolerance to Cd [11,12,13,14,15,16,17]. AtPDR11 relates to paraquat tolerance and AtABCG34 contributes to defense against necrotrophic pathogens by mediating the secretion of camalexin [18,19,20].

At present, approximately 50% of arable soil in the world is acidic, and the degree of soil acidification is further intensified. Aluminum toxicity is one of the most important factors limiting crop yield in acidic soil [21,22]. To adapt to this acidic soil environment, plants have formed corresponding detoxification mechanisms in the long-term historical evolution process. At present, aluminum resistance has two mechanisms. One is aluminum efflux detoxification, and the other is aluminum tolerance detoxification [23]. Regardless of which mechanism, we can see that transmembrane transporters play an important role in the detoxification of aluminum [24,25,26]. According to research, ABC transporters facilitate the detoxification of aluminum in plants. In *Arabidopsis*, AtALS3 (AtABCI16), which is involved in plant tolerance to aluminum toxicity, contains only a TMD domain and is an ABC transporter located in the plasma membrane [27]. When interacting with its interactive protein, AtSTAR1 (AtABCI17), containing NBD domain, forms an ABC transporter complex located in the tonoplast and represses STOP1 protein accumulation in the nucleus to inhibit the STOP1-ALMT1 pathway [28,29]. STOP1 directly binds to the *ALMT1* promoter to improve plant tolerance to aluminum [30]. AtABCB27 (AtALS1) possibly also chelates Al [31]. OsSTAR1/OsSTAR2 and FeSTAR1/FeSTAR2 also are required for Al tolerance in rice and buckwheat, respectively [32,33,34]. AtALS1, a half-type ABC transporter, is located in the vacuolar membrane of root cells and is required for root growth in an aluminum-toxic environment by facilitating the vacuolar sequestration of Al [31,35]. OsALS1 (OsABCB25), a homologous protein of AtALS1, is also located in the vacuolar membrane and is involved in the internal detoxification of Al in rice [36]. In wheat, *TaMDR1* has been reported to be involved in Al toxicity [14]. FeALS1.1 and FeALS1.2 are required for the internal detoxification of aluminum by sequestering Al into the vacuoles in buckwheat [25,37].

Soybean (*Glycine max* (L.) Merr.) is an important oil crop worldwide. Aluminum toxicity can seriously affect the growth and physiology of soybean, such as in the Rhizobium-legume symbiosis system, which is the key system of agricultural nitrogen cycle balance. Plant genotype is also an important factor to establish an effective nodulation and nitrogen fixation under Al stress [38]. However, research on the relationship between ABC transporters and aluminum tolerance in soybean is lacking. Therefore, this study utilized a publicly available whole genome sequence [39] to identify all the putative ABC transporters in soybean. Their phylogenetic relationship, expression patterns, structures of genes and proteins, gene duplication, gene collinearity, and chromosomal distribution were also analyzed. Furthermore, soybean ABC transporter genes related to aluminum detoxification were further investigated by differential gene expression analysis. This study provides useful clues for the further understanding of the biological functions of ABC transporters in soybean.

## 2. Results

### 2.1. Genome-Wide Identification of Soybean ABC Transporters

**Figure 1:**
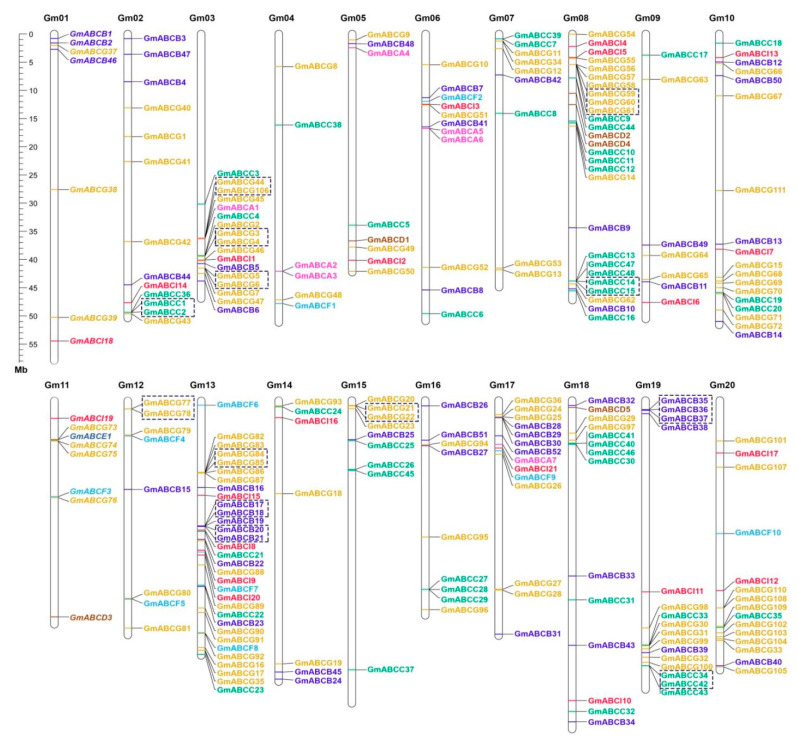
Chromosomal distribution of soybean ABC genes. The chromosome number is indicated at the top of each bar. The scale on the left represents the length in megabases (Mb). The tandemly duplicated GmABC genes are shown in black boxes.

A total of 255 GmABC protein sequences were identified in the genome of soybean and then classified into eight subfamilies. These genes were denoted as *GmABCA1*-*GmABCA7*, *GmABCB1*-*GmABCB52*, *GmABCC1*-*GmABCC48*, *GmABCD1*-*GmABCD5*, *GmABCE1*, *GmABCF1*- *GmABCF10*, *GmABCG1*-*GmABCG111*, and *GmABCI1*-*GmABCI21*, according to their position in the chromosome (Figure 1). Basic information on the *GmABC* genes based on their subfamilies, including gene ID number, number of amino acid (aa) residues, domain, gene position, molecular weight (kDa), isoelectric point (pIs), and subcellular localization, is shown in Table S1. The proteins ranged from 127 to 1897, and molecular weight varied from 13.51 kDa to 211.71 kDa. Their pIs ranged from 5.10 to 9.88. Approximately 70% of GmABC proteins was predicted to be located in the plasma membrane, and many of them were in the chloroplast and cytoplasm. A few of them were the mitochondrion, vacuole, nucleus, endoplasmic reticulum, and Golgi apparatus.

### 2.2. Phylogenetic Analysis of the GmABC Family

**Figure 2:**
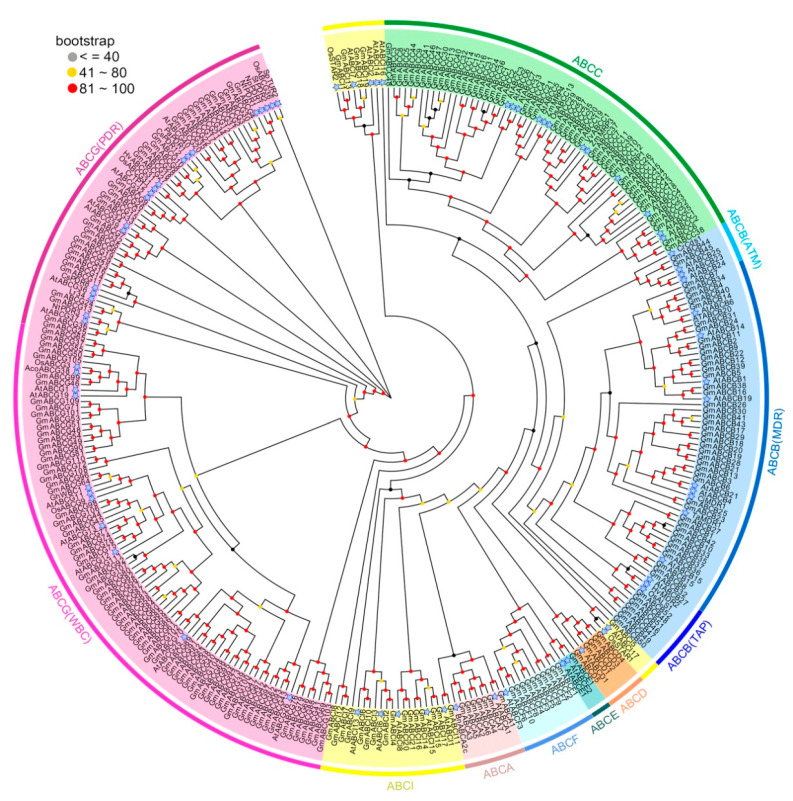
Phylogenetic tree of ABC transporter proteins from soybean. The unrooted phylogenetic tree was constructed by MEGA 7.0 using the maximum likelihood (ML) algorithm with 1000 bootstraps. Eight subfamilies are highlighted by different colors.

To better understand the evolutionary relationship of the GmABC proteins further, we constructed a phylogenetic tree by using the GmABCs’ amino acid sequences and the 77 ABC transporters in plants with known functions, which had been previously reported (Figure 2, Table S2). Similar to those in *Arabidopsis* and rice, the GmABC proteins in soybean were also divided into eight subfamilies (ABCA—ABCG and ABCI) (Figure 2, Table S1). Among the 255 GmABC proteins, subfamily ABCG ranked first and accounted for 43.5%; ABCB and ABCC came in second, and ABCE was the least common. Subfamily ABCG was divided into two clades: clade PDR (36 members) and clade WBC (75 members); subfamily ABCB was divided into three clades: clade MDR, clade ATM, and clade TAP, containing 43, 2, and 7 members, respectively (Figure 2). In summary, this classification is consistent with the perspective of phylogenetic branch and classification criteria in other species.

### 2.3. Gene Structure and Conserved Motif Analysis

**Figure 3:**
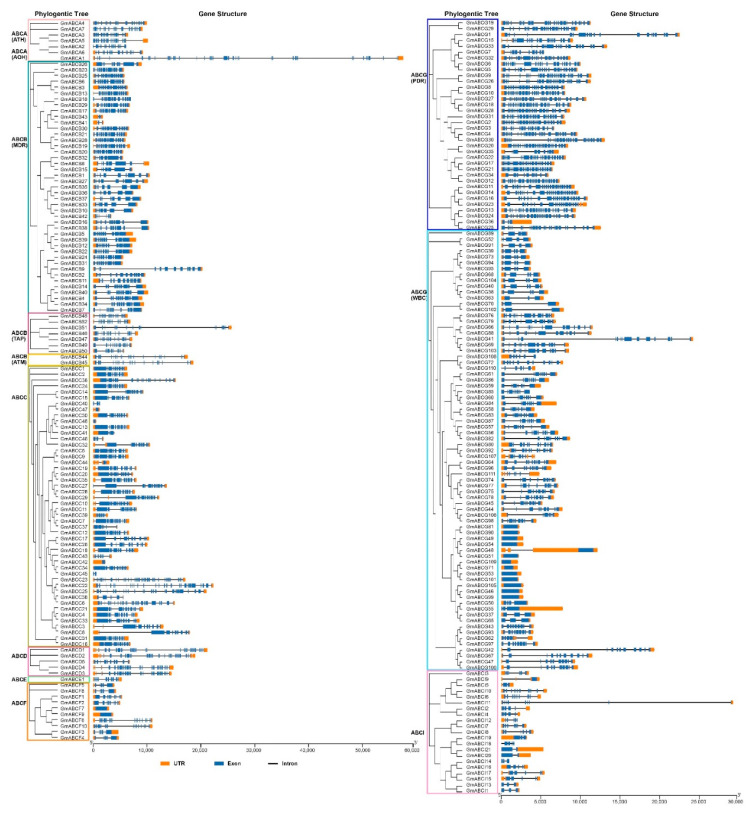
Gene structures of subfamilies GmABCA—GmABCG in soybean. Orange boxes, blue boxes, and black lines indicate UTRs, exons, and introns, respectively. The exon and intron length are calculated by the scale at the bottom.

The gene structure and conserved motif organization of GmABC were analyzed to characterize the structural diversity of GmABC genes further (Figure 3 and Figure S1). We found that GmABCs showed diversification between and within subfamilies through gene structural analysis. In terms of the distribution of exons, most GmABC transporters had 1–27 exons, and numerous exons were observed in GmABCC23 (36) and GmABCA1 (40). The number of exons was highly conserved on the same branches, especially the nearby protein-coding genes that have the same gene structure. Similarly, the number of introns among the different family genes also varied markedly (Figure 3). Specifically, 15 GmABC transporter genes, including *GmABCC42*, *GmABCF7*, *GmABCF9*, *GmABCG81*-*GmABCG54*, and *GmABCG51*-*GmABCG99*, only had one exon and no intron. Except for *GmABCI18*, *GmABCI14*, *GmABCG85*, *GmABCA2*, *GmABCB18*, *GmABCC40*, and *GmABCC45*, most GmABCs had UTR, including 97.25% (2248/255) of GmABCs.

**Figure 4:**
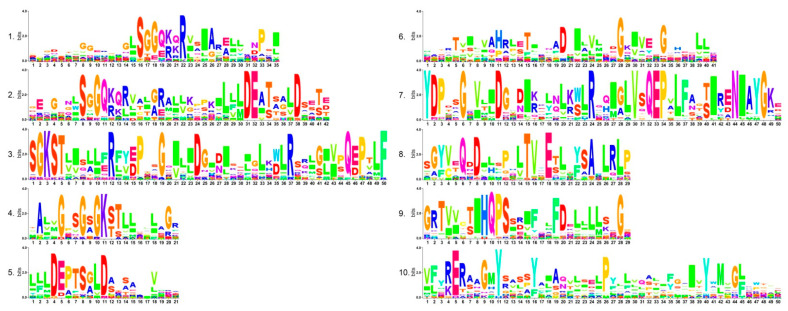
Conserved motifs of ABC transporter proteins in soybean. The conserved motifs of the GmABC proteins were elucidated using the MEME tool.

To gain the conserved domain of GmABCs, we analyzed them by using the MEME program. A total of 10 conserved motifs were predicted and comprised approximately 21–50 conserved amino acids (Figure 4, Table S3). As shown in Figure S1, GmABCs in the same branches displayed a similar motif order and composition as the gene structure. Both of them further supported the classification of subfamilies. Motifs -2, -4, -6, and -10 were prevalent in most GmABCs except in some GmABCGs, GmABCB, GmABCD, GmABCE, and GmABCF, respectively; motifs -1, -4, and -5 were arranged in the same order within most GmABCCs and GmABCGs, followed by motifs 6 and -9 separately. Common motifs occur in most GmABCs, and some members in separate subfamilies also contain their unique motifs; for instance, motif -7 only existed in the GmABCBs; motifs -8 and -9 basically only existed in GmABCA, GmABCG, GmABCD, and GmABCG, respectively, whereas motif -3 was mainly found in GmABCA—GmABCD. Interestingly, some putative GmABCs (e.g., GmABCI20) did not contain the above domain but contained the typical domain of ABC transporters while containing dozens of amino acids.

### 2.4. Chromosome Distribution

The 255 identified GmABC genes were unevenly located on the 20 chromosomes, and the distribution across the chromosomes varied widely (Figure 1). The chromosome Gm13 harbored the largest number of GmABCs (32 genes/~12.6%), followed by Gm08 (27 genes/~10.6%); Gm04 exhibited the smallest number with six genes (~2.6%) (Table S4). Chromosome Gm06, 02, 17, 18, 20, 19, 03, and 10 contained 11, 13, 14, 14, 15, 16, 17, and 18 GmABC genes, respectively. A total of seven genes each were distributed on chromosomes Gm01, Gm09, and Gm14; eight genes each were scattered on chromosomes Gm05, Gm11, and Gm12; nine genes each were present on chromosomes Gm07, Gm15, and Gm16. As reported in other studies, the most GmABC genes (~90%) were located in both ends of the chromosomes, in addition to Gm13.

### 2.5. Gene Duplication and Synteny Analysis

Gene duplication, especially tandem and segmental duplications, play an indispensable role in expanding the gene family during the evolutionary process. Thus, we analyzed the locations of GmABC duplicates in the soybean genome and observed four different types of gene duplications, including 17 proximal, 20 tandem, 41 dispersed, and 177 whole genome duplications (WGD) (segmental) (Table S5). None of the genes was a singleton. Hence, segmental duplication was the main reason for the expansion of the GmABC transporter.

**Figure 5:**
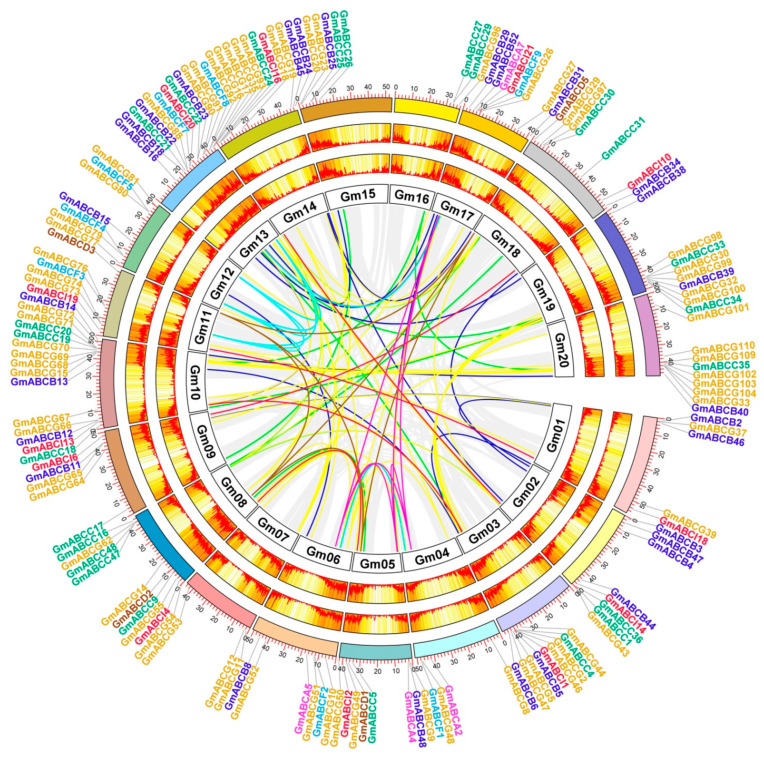
Schematic of the interchromosomal relationships of soybean ABC genes. Gray lines indicate all syntenic blocks in the soybean genome, and the colored lines indicate duplicated ABC gene pairs. The heatmaps represent the gene density.

A total of 125 GmABC gene pairs were detected, of which 15 pairs were tandem duplications, and 110 were segmental duplications (Figure 1 and Figure 5, Table S6). Two groups, including six genes (*GmABCB35*, *GmABCB36*, and *GmABCB37*; *GmABCG59*, *GmABCG60*, and *GmABCG61*), each contained two tandem duplication gene pairs located on the same chromosome Gm08 and Gm19 and were adjacent to each other. Chromosomes Gm03, Gm08, Gm13, and Gm19, each contained three tandem duplication gene pairs, whereas Gm02, Gm12, and Gm15 all contained one (Figure 1).

To detect which selection type promoted GmABC transporter evolution, we calculated Ka/Ks values for GmABC genes in duplicated genes. In addition to the *GmABCA2*/*GmABCA5*, *GmABCA4*/*GmABCA5*, and *GmABCA5*/*GmABCA7* gene pairs, the Ka/Ks ratios of all GmABC gene pairs were less than 1, suggesting that intense purifying selection was accompanied by the evolution of GmABC genes (Table S6). In the *GmABCA2*/*GmABCA5*, *GmABCA4*/*GmABCA5*, and *GmABCA5*/*GmABCA7* gene pairs, most of the synonymous mutation sites occurred. That is, the sequence divergence was large and the evolutionary distance was long. The divergence times of GmABC gene pairs were also evaluated. The divergence time of GmABC tandem duplication pairs ranged from 4.2 MYA to 120.5 MYA, with an average time of 28.8 MYA, whereas the segmental duplication pairs diverged between 5.3 and 106.6 MYA with an average time of 27.9 MYA (Table S6). In addition, the *GmABCG77*/*GmABCG78* gene pair might have occurred at approximately 120.5 MYA, which is an ancient event. Since then, no tandem duplication event occurred in 65 MYA until 54.5 MYA. However, segmental duplication events arose nearly 13 MYA later than tandem duplication and occurred all the time. The result showed that tandem duplications have a relatively earlier origin (Table S6).

**Figure 6:**
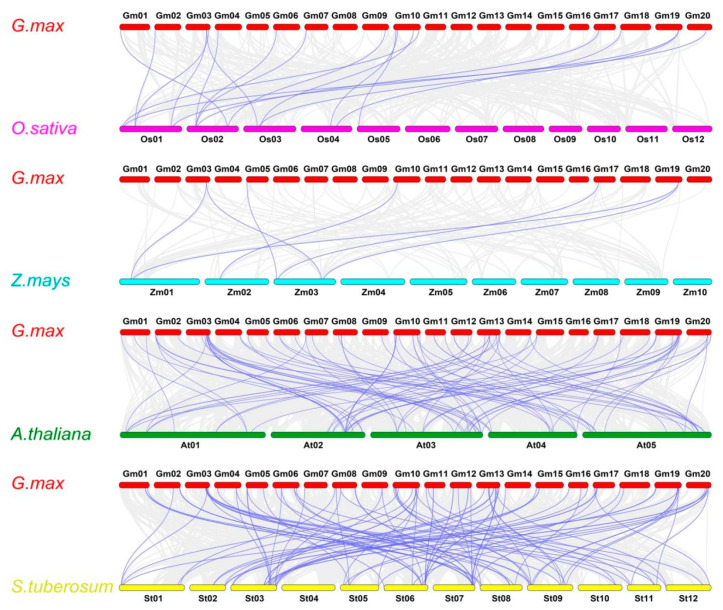
Collinearity analysis of ABC transporter genes between soybean and four other species (*A. thaliana*, *O. sativa*, *S. tuberosum*, and *Z. mays*). The gray curved lines represent the collinear blocks in the whole genome of soybean and other plants, the four species. The purple curved lines represent syntenic ABC transporter gene pairs.

To better understand the evolutionary origin of the GmABC gene family further, we analyzed the genome-wide collinearity of soybean with three other representative species and constructed a collinearity plot (Figure 6). A total of 88, 18, 98, and 7 collinear gene pairs were identified in *A. thaliana*, *O. sativa*, *S. tuberosum*, and *Z. mays*, respectively (Table S7). We also found the syntenic genes of GmABCs locating on almost all chromosomes of *A. thaliana* and *S. tuberosum*, but only on a few chromosomes of *O. sativa* and *Z. mays*. In addition, more collinearity blocks at the genome scale were found between soybean and *A. thaliana* and between soybean and *S. tuberosum* (Figure 6), indicating that the GmABCs have remained closely related to *A. thaliana* and *S. tuberosum* during evolution. The Ka/Ks ratios of the orthologous gene pairs were calculated and the Ka/Ks values of all ortholog pairs were below 0.5, confirming that the GmABC family across the species was under a stabilizing or strong purifying selection during its evolution (Table S7). Interestingly, several collinear relationships were found in different species, and some collinear gene pairs were available in dicotyledonous plants (soybean and *A. thaliana*/*S. tuberosum*) but absent in the monocotyledonous ones (sugarcane and *O. sativa*/*Z. mays*). For example, 74 GmABCs and 46 AtABCs had a collinear relationship, and one-to-one matches had the most relationships, such as *GmABCG39*/*AT1G31770.1*. Many-to-one cases and one-to-many matches, such as (*GmABCG12*, *GmABCG17*, *GmABCG20*)/ *AT1G15520.1* and *GmABCG54*/ (*AT2G47000.5*, *AT3G62150.2*, *AT4G01830.1*), also existed (Table S7), indicating that GmABCs were relatively conserved and that collinear GmABCs between species may have originated from the same ancestor.

### 2.6. Expression Pattern of GmABC Genes in Different Tissues

**Figure 7:**
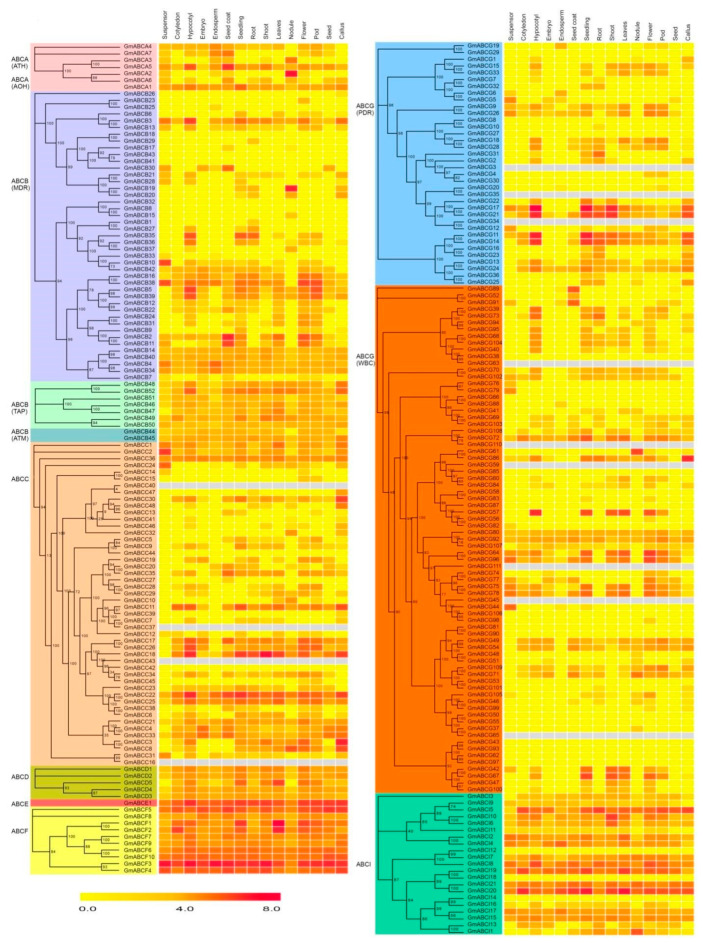
Expression patterns of ABC transporter genes in developmental stages and tissues of soybean. The color scale bars on the bellow display the expression levels (median log_2_ (TPM+1)) of each gene. Red represents high expression levels, whereas yellow represents low expression levels.

**Figure 8:**
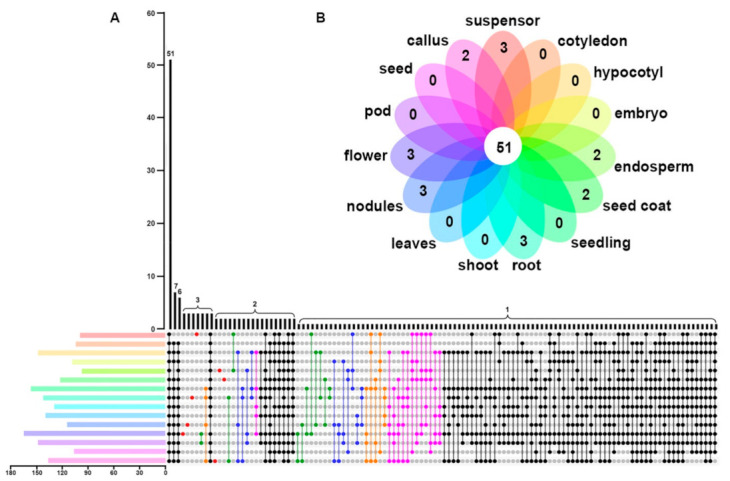
Distribution of tissue-independent and tissue-specific genes in soybean. (**A**) The UpSet plot depicts how many *GmABCs* are expressed in each tissue (suspensor, cotyledon, hypocotyls, embryo, endosperm, seed coat, seedling, root, shoot, leaves, nodule, flower, pod, seed, and callus). Each color represents a tissue, and its length refers to the number of expressed genes. The black dots indicate the number of genes expressed in each tissue. The only dot (red) represents the number of genes unique to this tissue, and two or more dots connected by a line represent the number of genes expressed in several tissues. The green, blue, golden yellow, and pink lines connecting the dots represent the number of genes expressed in two, three, four, and five tissues, respectively. The dark line represents the number of genes expressed in at least six tissues. (**B**) The Venn diagram shows the specific expressed *GmABCs* in different tissues of soybean.

To better understand the biological functions of the identified genes further, we downloaded the transcriptional data of fifteen soybean tissues from a publicly available gene expression browser website and analyzed the expression patterns (Table S8). As shown in Figure 7, the GmABCs exhibited different expression levels in the suspensor, cotyledon, hypocotyls, embryo, endosperm, seed coat, seedling, root, shoot, leaves, nodule, flower, pod, seed, and callus. For example, a total of seven genes in GmABCA were all expressed in the suspensor and seed coat, among which *GmABCA1* and *GmABCA5* were expressed in all tissues, indicating that the GmABCA members might have multiple biological functions. GmABCB, GmABCC, and GmABCG exhibited that some members were expressed in almost all tissues, and some were almost not expressed. However, almost all the members of GmABCD, GmABCE, GmABCF, and GmABCI showed relatively high expression in all tissues, especially GmABCE and GmABCF. Most homologous gene pairs broadly exhibited similar expression patterns, but there were some exceptions. For example, the gene pair *GmABCG10*/*GmABCG27* exhibited no expression in all tested tissues, and the gene pair *GmABCG10*/ *GmABCG18*, *GmABCG18* exhibited relatively high expression in almost all tissues (Figure 7). Among the GmABC genes examined, 51 were expressed in all fifteen tissues, 29 were not expressed in the tissue samples, and 18 were expressed only in the single tissue that showed tissue specificity (Tables S8 and S9, Figure 8A,B).

### 2.7. Expression of GmABC Genes in Response to Al Toxicity

**Figure 9:**
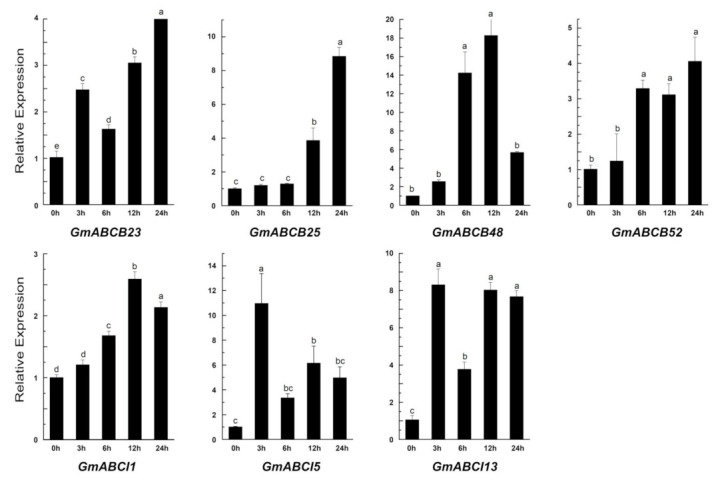
Time-dependent expression of seven *GmABC* gene expressions. The roots of soybean exposed to a 0.5 mM CaCl_2_ solution (pH 4.5) containing 50 μM AlCl_3_ for 0, 3, 6, 12, and 24 h were used for analysis. The expression was determined by qRT-PCR and β-tubulin was used as an internal control. The treatment without AlCl_3_ (0 μM AlCl_3_) was used for control. All data shown are means ± SD of three biological replicates. Bars with different lowercase letters indicate significant differences (*p* < 0.05).

According to the phylogenetic tree, we chose *GmABCB23*, *GmABCB25*, *GmABCB48*, *GmABCB52*, *GmABCI1*, *GmABCI5*, and *GmABCI13* for further gene expression analysis by qRT-PCR (Figure 9). These genes were chosen because they were on the same evolutionary branch as genes with known functions related to aluminum toxicity. Therefore, we analyzed and then performed qRT-PCR to identify their expression patterns related to aluminum toxicity. After treatment, the expression of all selected genes was induced by Al in roots, but the induced expression was different. *GmABCB25* and *GmABCB52* were upregulated; *GmABCB25*, *GmABCB48*, and *GmABCI1* were first upregulated and then downregulated; *GmABCB23* and *GmABCI13* were first upregulated and then downregulated, and finally recovered to a high level (Figure 9).

## 3. Discussion

The ABC transporter gene family is one of the largest and oldest known protein families. In plants, ABC transporters have many types, complex structures, and diverse functions, which are involved in all the life activities of plants. In previous studies, the ABC transporter gene family has been systematically analyzed in multiple plants and has been found to play vital roles in the growth, development, and response to abiotic and biotic stresses of plants [6,40,41,42,43]. Here, we performed a comprehensive analysis of the GmABC genes and provided a reference for further exploration in soybean.

In our study, a total of 255 putative *GmABC* genes were identified in soybean via genome-wide analysis, accounting for 0.296% of the total 86,247 annotated genes of its reference sequence (Table S1). Reports have shown the existence of 154 *SlABCs* [6], 121 *OsABCs* [41], 120 *AtABCs* [41], 130 *ZmABCs* [42], 121 *StABCs* (unpublished), and 191 *MdABCs* [40]. Genome size varies in different plants (i.e., *G. max* [1.1 Gb], *S. lycopersicum* [942 Mb], *O. sativa* [466 Mb], *A. thaliana* [125 Mb], *Z. mays* [2.3 Gb], *S. tuberosum* [844 Mb], *M. domestica* [742 Mb]), indicating that the number of ABC transporters is inconsistent with their genome sizes. However, ABC transporter numbers in soybean are higher than those in all the other known species. Soybean has undergone several ancestral WGD events in the long history of evolution, and ~60% sequences are repetitive. Approximately 75% of the genes are multi-copy genes, and ~25% of soybean genes have experienced two times of polyploidization, and their structure and function are highly conserved [39,44,45], probably leading to the greater number of ABC genes in soybean compared with other species. During the evolutionary history of the GmABC gene family, gene diversification resulted in huge differences in the length and type of amino acid. Thus, the protein characteristics are remarkably different, including the MWs, isoelectric points and localization. For example, most GmABC proteins are predicted to be located in the plasma membrane, followed by the chloroplast (Table S1).

The ten conserved motifs of GmABC proteins were also identified and analyzed (Figure 4 and Figure S1, Table S3) and were found to be basically the same as that of *Brassica rapa,* tomato, and flax [6,46,47]. This result indicates that GmABCs may have similar functional characteristics. At the same time, we found that each subfamily has unique motifs, which were in a different order according to the analysis of the motif position of GmABC proteins (Figure S1). This finding suggests that each subfamily has specific functions [48]. Putative functions of conversed motifs were shown in Table S3 by ScanProsite analysis, and basically all of them were related to the ABCB and ABCG subfamily. Some GmABCs have no conserved motifs and only contain dozens of amino acids coding for the typical domain of ABC transporters, indicating that the GmABC family may be expanding and is possibly caused by WGD. In the evolutionary history, many higher plants underwent several WGD events [44] and polyploidization. For species that experienced WGDs, the subsequent WGD event would accelerate the gene loss of the previous WGD. The genes retained after WGDs could explain how the duplicated genes generated by WGD contributed to the organism’s evolution and enhanced their adaption to the environment [49,50,51,52]. The dozens of amino acids coding for the typical domain of ABC transporters may provide some clues to the origin and evolution of the GmABC gene family.

WGDs, which serve as evolutionary switches, play vital roles in the development of different plant species and their adaptation to various environments. Moreover, gene duplications are an important source of power in gene diversification and genomic evolution [53,54]. Similar to various plants [47,55,56,57], WGD/segmental duplications (69.41%) contribute the most to the expansion of GmABC gene families in soybean (Table S5), suggesting the high conservation of the GmABC family. Synteny analysis results showed that the Ks values and the number of homologous gene pairs between dicotyledons were much higher than that in monocotyledons (Table S7). The same is true for collinearity blocks (Figure 6). The above results indicated that a close relationship with ABC genes existed among dicotyledonous plants and supported the view that dicotyledons were more ancient than monocotyledons. It also suggested that the ABC family mainly expanded during the evolution of dicotyledons. The gene duplication results revealed that 125 GmABC gene pairs were duplicated genes. We found that the span of the Ks value and the number of segmental duplicates (0.07–1.38, 110) were larger than those in tandem duplicates (0.05–1.57, 15) (Table S6), indicating that the segmental duplication events ran through the whole evolutionary history of soybean. It also showed that the GmABC expansion originated from different periods over the course of soybean evolution through the evaluated divergence times of the duplication events (Table S6). Syntenic analysis and gene duplication *authenticated that* GmABC genes underwent strong purifying selection. Our results are close to those in other plants [47,58], indicating that GmABC genes are relatively conservative in the process of evolution and maintain functional stability. According to the expression profiles of *GmABCs* in different tissues, the majority of *GmABCs* differed in their expression patterns, and the minority displayed similarities (Figure 7). These phenomena further explained the functional diversity and expression-site specificity of GmABC genes. Therefore, further experiments are needed to obtain whether functional differentiation occurred.

Many reported studies have shown that ABC transporters are involved in abundant functions, including the transport of secondary metabolites, antibiotics, drugs, carbohydrates, lipids, and ions, as well as heavy metal detoxification [59,60,61,62,63,64]. Aluminum toxicity, which is a global agricultural problem, can limit crop productivity by inhibiting root growth. In recent years, the progress of genome sequence research has provided convenience for the cloning and identification of genes related to Al tolerance in plants [26,65,66,67], e.g., AtALS3, AtSTAR1, OsALS1, FeALS1.1, FeALS1.2, FeSTAR2, FeSTAR1, OsSTAR1, and OsSTAR2 [32,33,36,37]. Taken together, these research results indicate that ABC transporters play important roles in plant resistance to Al toxicity. However, whether this internal detoxification mechanism exists in soybean and which gene is responsible for this mechanism have yet to be investigated. The soybean GmABCs’ functions could be inferred on the basis of phylogenetic trees of GmABCs and 77 known plant ABC transporters (Figure 2). For example, GmABCB44, GmABCB45, and three known ABC transporter proteins (i.e., AtABCB23, AtABCB24, and AtABCB25, all of which are known as modulating Fe-S cluster biogenesis) [68] are located on the same branch of the phylogenetic tree (Figure 2, Table S2), suggesting that GmABCB44 and GmABCB45 may also be involved in modulating Fe-S cluster biogenesis. Thus, we selected *GmABCB23*, *GmABCB25, GmABCB48, GmABCB52, GmABCI1, GmABCI5*, and *GmABCI13* as candidate genes (Figure 2) according to previous genes related to aluminum detoxification [14,31,32,36,61]. We found that the aluminum toxicity considerably enhanced the candidate genes’ expressions (Figure 9), indicating that they might be involved in Al toxicity. GmABCB48, GmABCB52, OsALS1, and AtALS1, which are located in the same evolutionary branch, suggest that GmABCB48 and GmABCB52 are likely to be located in the tonoplast and are responsible for the sequestration of Al into the vacuoles [31,35,36]. GmABCI5 (homolog of AtSTAR1 and OsSTAR1) may interact with GmABCI1 or GmABCI13 (homolog of AtALS3 and OsSTAR2) and form an ABC transporter to regulate Al tolerance through the vesicular transport of UDP-glucose, which affects hemicellulose metabolism by regulating XET activity [32,33,34]. Whether they work similar to known genes must be determined for validation. Our research provides the potential roles of GmABC transporters in Al toxicity and a foundation for further functional characterization.

## 4. Materials and Methods

### 4.1. Identification of Putative ABC Proteins in Soybean

Information on the whole soybean genome sequence, protein sequence, and gene annotation (Wm82.a4.v1) was downloaded from the Phytozome v12.1 database (https://phytozome.jgi.doe.gov/pz/portal.html, last accessed 12 April 2020). To identify soybean ABC protein candidates, the Hidden Markov Model (HMM) analysis was used for the search. From UniPort and RGAP, all ABC protein sequences of *Arabidopsis* and rice were downloaded as seed sequences to establish HMM profiles [69,70,71]. On the basis of this model, HMMER^3.0^ software was used to search all ABC proteins in the soybean genome [72]. The identified ABC proteins from soybean were also filtered and validated by the presence of conserved domains (Pfam: PF00005; Pfam: PF01061; Pfam: PF00664; Pfam: PF01458; Pfam: PF02470) using the Pfam (http://pfam.xfam.org/, last accessed 8 May 2020) and the Simple Modular Architecture Research Tool (SMART, http://smart.embl-heidelberg.de/smart/batch.pl, last accessed 8 May 2020). A total of 255 soybean ABC proteins were identified.

The members of the soybean ABC family were named in accordance with their chromosome position. The number of amino acids, theoretical molecular weight (MW), and theoretical isoelectric point (pI) of the soybean ABC family were obtained by ExPASy [73]. Their subcellular localization was predicted by WoLFPSORT (https://www.genscript.com/wolf-psort.html?src=leftbar, last accessed 12 June 2020). The Phytozome database could extract the information on ABC genes, including their chromosomal distribution and their start and end positions on the chromosomes.

### 4.2. Gene Structure, Conversed Motif, and Phylogeny Analysis

The software TBtools (ver 1.0692) was used to draw the gene structures by comparing the cDNA sequences and its corresponding genomic DNA sequences of ABC transporter members [74,75]. The amino acid sequences were analyzed by MEME Suite v5.2.0, and the conserved motifs were identified [76]. The parameters were set as the default value of the software, and the number of motifs was 10. The discovered motifs were searched in the Expasy-Prosite database with ScanProsite server (https://prosite.expasy.org/scanprosite/, last accessed 11 July 2020) [77].

All the protein sequences of 255 soybean ABC proteins and 77 previously reported ABC transporters from other plant species were used for multiple sequence alignments by MAFFT 7.0 (https://mafft.cbrc.jp/alignment/server/, last accessed 11 July 2020). The unrooted phylogenetic tree was then constructed by MEGA 7.0 using the ML algorithm with 1000 bootstraps. The result of the ML tree was viewed and edited by Evolview [78].

### 4.3. Chromosomal Locations, Gene Duplication, and Gene Collinearity Analysis

The chromosomal locations of GmABC genes were illustrated by MapInspect. Gene duplication events and genome collinearity were analyzed by the MCScanX program in TBtools and visualized in CIRCOS using default parameters. The syntenic relationship of ABC genes was determined by Dual Synteny Plotter software in TBtools. The putative duplication events were detected for the ABC genes. To evaluate the selection pressure, the ratios of non-synonymous (Ka) substitutions to synonymous (Ks) substitutions of each duplicated ABC gene were calculated by the NG method of TBtools. Ks values > 2.0 must be discarded to avoid the saturation of substitutions [79,80]. The occurrence time of duplicated GmABC gene pairs was calculated by “T = Ks/(2λ × 10^−6^)” (λ = 6.5 × 10^−9^) [81].

### 4.4. Expression Pattern Analysis of the GmABC Genes

The expression data of all the 255 *GmABC* genes in fifteen different tissues (suspensor, cotyledon, hypocotyls, embryo, endosperm, seed coat, seedling, root, shoot, leaves, nodule, flower, pod, seed, and callus) of soybean were obtained from the web interface (http://venanciogroup.uenf.br/resources/, last accessed 23 August 2020) to detect the expression profile [82]. The gene expression was calculated and normalized in TPM (Transcripts Per Million) using StringTie (*–e* option) [67]. Lastly, all the median TPMs per tissue were used to make heatmaps through TBtools to investigate their expression patterns.

### 4.5. Plant Materials and QRT-PCR Analysis

The soybean (*Glycine max* L.) seeds from our lab were used for analysis and the growth conditions are previously described [11]. The uniform seedlings were selected and exposed to 0.5 mM CaCl_2_ solution (pH 4.5) containing either 0 μM AlCl3 (control) or 50 μM AlCl_3_ (treatment) for 3, 6, 12, and 24 h, respectively. Then, the collected root tips (1 cm) were quickly frozen in liquid nitrogen and stored at −80 °C for subsequent RNA extraction. The experiment was performed in triplicate.

All the samples’ total RNA were extracted by using *Trizol* reagent (Invitrogen, Carlsbad, CA, USA), as described by the manufacturer’s protocol. The cDNAs were synthesized by *Prime Script* reverse transcriptase (TaKaRa, Kusatsu, Shiga, Japan). The number of transcripts of the selected *GmABCs*and gene *β-tubulin* (internal control) were determined by using SYBR Premix Ex Taq™ II kit (TaKaRa, Kusatsu, Shiga, Japan). qPCR conditions were as follows: stage 1, 95 °C for 5 min; stage 2, 40 cycles of 10 s at 95 °C, 30 s at 60 °C; stage 3, 95 °C for 15 s, 60 °C for 60 s, 95 °C for 15 s. Stage 3 was for melting curve analysis. The formula 2^−ΔΔCT^ was utilized to calculate the relative expression levels. The *β-tubulin* was used to normalize the expression levels of the genes detected in this study. The gene-specific primers used for this analysis are shown in Table S10.

## Data Availability

The whole soybean genome sequence, protein sequence, and gene annotation information (Wm82.a4.v1) were available in the phytozome v12.1 database (https://phytozome.jgi.doe.gov/pz/portal.html, last accessed 12 April 2020). The expression data of all the 255 *GmABC* genes in fifteen different tissues of soybean were obtained from the web interface (http://venanciogroup.uenf.br/resources/, last accessed 23 August 2020) and included in Table S7 of this article.

## Supplementary Materials

The following are available online at https://www.mdpi.com/article/10.3390/ijms22126556/s1. Figure S1. Conserved motifs in GmABC proteins. The 10 conversed motifs were identified by MEME and displayed by 10 various different colors. Table S1. Basic information about the ABC transporter gene family in soybean. Table S2. The 77 known ABC transporters in plants. Table S3. Conserved motifs present in ABC transporter in soybean. Table S4. Chromosome distribution of GmABC superfamily genes in soybean genome. Table S5. The duplicated type of *GmABC* genes in soybean. Table S6. KaKs calculation and estimated divergence time for each pair of segmentally and tandemly duplicated *GmABC* gene pairs. Table S7. One-to-one orthologous relationships between *G.max* and other four plant species. Table S8. RNA-seq data of 242 soybean ABC genes in fifteen tissues as shown in median TPM (transcripts per million). Table S9. List of highly expressed ABC transporter genes in different tissues. Table S10. Primers used for QRT-PCR.

## References

[1] Theodoulou F.L., Kerr L.D. (2015). ABC transporter research: Going strong 40 years on. Biochem. Soc. Trans..

[2] Higgins C.F., Linton K.J. (2004). The ATP switch model for ABC transporters. Nat. Struct. Mol. Biol..

[3] Zhu L., Xu J., Zhang D.B. (2012). Molecular evolution, expression and functional network prediction analysis of ABC transporter gene family in *Arabidopsis thaliana*. Plant Physiol. J..

[4] Nguyen V.N., Moon S., Jung K.H. (2014). Genome-wide expression analysis of rice ABC transporter family across spatio-temporal samples and in response to abiotic stresses. J. Plant Physiol..

[5] Zhang X.D., Zhao K.X., Yang Z.M. (2018). Identification of genomic ATP binding cassette (ABC) transporter genes and Cd-responsive ABCs in *Brassica napus*. Gene.

[6] Ofori P.A., Mizuno A., Suzuki M., Martinoia E., Reuscher S., Aoki K., Shibata D., Otagaki S., Matsumoto S., Shiratake K. (2018). Genome-wide analysis of ATP binding cassette (ABC) transporters in tomato. PLoS ONE.

[7] Schaedler T.A., Thornton J.D., Kruse I., Schwarzländer M., Meyer A.J., van Veen H.W., Balk J. (2014). A conserved mitochondrial ATP-binding cassette transporter exports glutathione polysulfide for cytosolic metal cofactor assembly. J. Biol. Chem..

[8] Davies T.G.E., Theodoulou F.L., Hallahan D.L., Hallahan D.L., Forde B.G. (1997). Cloning and characterization of a novel p-glycoprotein homologue from barley. Gene.

[9] Shitan N., Bazin I., Dan K., Obata K., Kigawa K., Ueda K., Sato F., Forestier C., Yazaki K. (2003). Involvement of CjMDR1, a plant multidrugresistance-type ATP-binding cassette protein, in alkaloid transport in *Coptis japonica*. Proc. Natl. Acad. Sci. USA.

[10] Park J., Song W.-Y., Ko D., Eom Y., Hansen T.H., Schiller M., Lee T.G., Martinoia E., Lee Y. (2012). The phytochelatin transporters ATABCC1 and ATABCC2 mediate tolerance to cadmium and mercury. Plant J..

[11] Bessire M., Borel S., Fabre G., Carraca L., Efremova N., Yephremov A., Cao Y., Jetter R., Jacquat A.C., Metraux J.P. (2011). A member of the pleiotropic drug resistance family of ATP binding cassette transporters is required for the formation of a functional cuticle in *Arabidopsis*. Plant Cell.

[12] Chen G., Komatsuda T., Ma J.F., Nawrath C., Pourkheirandish M., Tagiri A., Hu Y.G., Sameri M., Li X., Zhao X. (2011). An ATP-binding cassette subfamily G full transporter is essential for the retention of leaf water in both wild barley and rice. Proc. Natl. Acad. Sci. USA.

[13] Fu S., Lu Y., Zhang X., Yang G., Chao D., Wang Z., Shi M., Chen J., Chao D.-Y., Li R. (2019). The ABC transporter ABCG36 is required for cadmium tolerance in rice. J. Exp. Bot..

[14] Sasaki T., Ezaki B., Matsumoto H. (2002). A gene encoding mutidrug resistance (MDR)-like protein is induced by aluminium and inhibitors of calcium flux in wheat. Plant Cell Physiol..

[15] Hanikenne M., Motte P., Wu M.C.S., Wang T., Loppes R., Matagne R.F. (2005). A mitochondrial half-size ABC transporter is involved in cadmium tolerance in *Chlamydomonas reinhardtii*. Plant Cell Environ..

[16] Gaillard S., Jacquet H., Vavasseur A., Leonhardt N., Forestier C. (2008). AtMRP6/AtABCC6, an ATP-binding cassette transporter gene expressed during early steps of seedling development and up-regulated by cadmium in *Arabidopsis thaliana*. BMC Plant Biol..

[17] Brunetti P., Zanella L., De Paolis A., Di Litta D., Cecchetti V., Falasca G., Barbieri M., Altamura M.M., Costantino P., Cardarelli M. (2015). Cadmium-inducible expression of the ABC-type transporter AtABCC3 increases phytochelatin-mediated cadmium tolerance in *Arabidopsis*. J. Exp. Bot.

[18] Xi J., Xu P., Xiang C. (2012). Loss of AtPDR11, a plasma membrane-localized ABC transporter, confers paraquat tolerance in *Arabidopsis thaliana*. Plant J..

[19] Khare D., Choi H., Huh S.U., Bassin B., Kim J., Martinoia E., Sohn K.H., Paek K.-H., Lee Y. (2017). Arabidopsis ABCG34 contributes to defense against necrotrophic pathogens by mediating the secretion of camalexin. Proc. Natl. Acad. Sci. USA.

[20] Yang G., Fu S., Huang J., Li L., Long Y., Wei Q., Wang Z., Chen Z., Xia J. (2021). The tonoplast-localized transporter OsABCC9 is involved in cadmium tolerance and accumulation in rice. Plant Sci..

[21] Gupta N., Gaurav S.S., Kumar A. (2013). Molecular basis of aluminium toxicity in plants: A Review. Am. J. Plant Sci..

[22] Nunes-Nesi A., Brito D.S., Inostroza-Blancheteau C., Fernie A.R., Araújo W.L. (2014). The complex role of mitochondrial metabolism in plant aluminum resistance. Trends Plant Sci..

[23] Ma J.F. (2007). Syndrome of aluminum toxicity and diversity of aluminum resistance in higher plants. Int. Rev. Cytol..

[24] Yokosho K., Yamaji N., Mitani-Ueno N., Shen R.F., Ma J.F. (2016). An Aluminum-Inducible IREG gene is required for internal detoxification of aluminum in buckwheat. Plant Cell Physiol..

[25] Lei G.J., Yokosho K., Yamaji N., Fujii-Kashino M., Ma J.F. (2017). Functional characterization of two half-size ABC transporter genes in aluminium-accumulating buckwheat. New Phytol..

[26] Wang H., Ji F., Zhang Y., Hou J., Liu W., Huang J., Liang W. (2019). Interactions between hydrogen sulphide and nitric oxide regulate two soybean citrate transporters during the alleviation of aluminium toxicity. Plant Cell Environ..

[27] Ryan P.R., Tyerman S.D., Sasaki T., Furuichi T., Yamamoto Y., Zhang W.H., Delhaize E. (2011). The identification of aluminium-resistance genes provides opportunities for enhancing crop production on acid soils. J. Exp. Bot..

[28] Dong J., Piñeros M.A., Li X., Yang H., Liu Y., Murphy A.S., Kochian L.V., Liu D. (2017). An Arabidopsis ABC transporter mediates phosphate deficiency-induced remodeling of root architecture by modulating iron homeostasis in roots. Mol. Plant.

[29] Wang X., Wang Z., Zheng Z., Dong J., Song L., Sui L., Nussaume L., Desnos T., Liu D. (2019). Genetic dissection of Fe-dependent signaling in root developmental responses to phosphate deficiency. Plant Physiol..

[30] Tokizawa M., Kobayashi Y., Saito T., Kobayashi M., Iuchi S., Nomoto M., Tada Y., Yamamoto Y.Y., Koyama H. (2015). Sensitive to proton rhizotoxicity1, calmodulin binding transcription activator2, and other transcription factors are involved in aluminum-activated malate transporter1 expression. Plant Physiol..

[31] Larsen P.B., Cancel J., Rounds M., Ochoa V. (2007). Arabidopsis ALS1 encodes a root tip and stele localized half type ABC transporter required for root growth in an aluminum toxic environment. Planta.

[32] Huang C.F., Yamaji N., Mitani N., Yano M., Nagamura Y., Ma J.F. (2009). A bacterial-type ABC transporter is involved in aluminum tolerance in rice. Plant Cell.

[33] Che J., Yamaji N., Yokosho K., Shen R.F., Ma J.F. (2018). Two genes encoding a bacterial-type ATP-binding cassette transporter are implicated in aluminum tolerance in buckwheat. Plant Cell Physiol..

[34] Xu J.M., Lou H.Q., Jin J.F., Chen W.W., Wan J.X., Fan W., Yang J.L. (2018). A half-type ABC transporter FeSTAR1 regulates Al resistance possibly via UDP-glucose-based hemicelluloses metabolism and Al binding. Plant Soil.

[35] Delhaize E., Ma J.F., Ryan P.R. (2012). Transcriptional regulation of aluminium tolerance genes. Trends Plant Sci..

[36] Huang C.F., Yamaji N., Chen Z., Ma J.F. (2012). A tonoplast-localized half-size ABC transporter is required for internal detoxification of aluminum in rice. Plant J..

[37] Xu J.M., Wang Z.Q., Jin J.F., Chen W.W., Fan W., Zheng S.J., Yang J.L. (2019). Festar2 interacted by festar1 alters its subcellular location and regulates al tolerance in buckwheat. Plant Soil.

[38] Artigas Ramírez M.D., Silva J.D., Ohkama-Ohtsu N., Yokoyama T. (2018). In vitro rhizobia response and symbiosis process under aluminum stress. Can. J. Microbiol..

[39] Schmutz J., Cannon S.B., Schlueter J., Ma J., Mitros T., Nelson W., Hyten D.L., Song Q., Thelen J.J., Cjeng J. (2010). Genome sequence of the palaeopolyploid soybean. Nature.

[40] Feng Y., Sun Q., Zhang G., Wu T., Zhang X., Xu X., Han Z., Wang Y. (2019). Genome-wide identification and characterization of ABC transporters in nine rosaceae species identifying MdABCG28 as a possible cytokinin transporter linked to dwarfing. Int. J. Mol. Sci..

[41] Garcia O., Bouige P., Forestier C., Dassa E. (2004). Inventory and comparative analysis of rice and *Arabidopsis* ATP-binding cassette (ABC) systems. J. Mol. Biol..

[42] Pang K., Li Y., Liu M., Meng Z., Yu Y. (2013). Inventory and general analysis of the ATP-binding cassette (ABC) gene superfamily in maize (*Zea mays L.*). Gene.

[43] Chen W.W., Xu J.M., Jin J.F., Lou H.Q., Fan W., Yang J.L. (2017). Genome-wide transcriptome analysis reveals conserved and distinct molecular mechanisms of Al resistance in buckwheat (*Fagopyrum esculentum Moench*) leaves. Int. J. Mol. Sci..

[44] Jiao Y., Wickett N.J., Saravanaraj A., Chanderbali A.S., Landherr L., Ralph P.E., Tomsho L.P., Hu Y., Liang H., Sotis P.S. (2011). Ancestral polyploidy in seed plants and angiosperms. Nature.

[45] Zhao N., Ding X., Lian T., Wang M., Tong Y., Liang D., An Q., Sun S., Jackson S.A., Liu B. (2020). The effects of gene duplication modes on the evolution of regulatory divergence in wild and cultivated soybean. Front. Genet..

[46] Yan C., Duan W., Lyu S., Li Y., Hou X. (2017). Genome-wide identification, evolution, and expression analysis of the ATP-binding cassette transporter gene family in *Brassica rapa*. Front. Plant Sci..

[47] Khan N., You F.M., Datla R., Ravichandran S., Jia B., Cloutier S. (2020). Genome-wide identification of ATP binding cassette (ABC) transporter and heavy metal associated (HMA) gene families in flax (*Linum usitatissimum L.*). BMC Genom..

[48] Jiang S.-Y., Jin J., Sarojam R., Ramachandran S. (2019). A comprehensive survey on the terpene synthase gene family provides new insight into its evolutionary patterns. Genome Biol. Evol..

[49] Badouin H., Gouzy J., Grassa C.J., Murat F., Staton S.E., Cottret L., Lelandais-Brière C., Owens G.L., Carrère S., Mayjonade B. (2017). The sunflower genome provides insights into oil metabolism, flowering and Asterid evolution. Nature.

[50] Schwager E.E., Sharma P.P., Clarke T., Leite D.J., Wierschin T., Pechmann M., Akiyama-Oda Y., Esposito L., Bechsgaard J., Bilde T. (2017). The house spider genome reveals an ancient whole-genome duplication during arachnid evolution. BMC Biol..

[51] Ren R., Wang H., Guo C., Zhang N., Zeng L., Chen Y., Ma H., Qi J. (2018). Widespread whole genome duplications contribute to genome complexity and species diversity in angiosperms. Mol. Plant.

[52] Zhikai L., Schnable J.C. (2018). Functional divergence between subgenomes and gene pairs after whole genome duplications. Mol. Plant.

[53] Clark J.W., Donoghue P.C. (2018). Whole-genome duplication and plant macroevolution. Trends Plant Sci..

[54] Segraves K.A. (2017). The effects of genome duplications in a community context. New Phytol..

[55] Khan N., Fatima F., Haider M.S., Shazadee H., Liu Z., Zheng T., Fang J. (2019). Genome-wide identification and expression profiling of the polygalacturonase (PG) and pectin methylesterase (PME) genes in grapevine (*Vitis vinifera L.*). Int. J. Mol. Sci..

[56] Shazadee H., Khan N., Wang J., Wang C., Zeng J., Huang Z., Wang X. (2019). Identification and expression profiling of protein phosphatases (PP2C) gene family in *Gossypium hirsutum L*. Int. J. Mol. Sci..

[57] Die J.V., Gil J., Millán T. (2018). Genome-wide identification of the auxin response factor gene family in *Cicer arietinum*. BMC Genom..

[58] Cannon S.B., Mitra A., Baumgarten A., Young N.D., May G. (2004). The roles of segmental and tandem gene duplication in the evolution of large gene families in *Arabidopsis thaliana*. BMC Plant Biol..

[59] Goodman C.D., Casati P., Walbot V. (2004). A Multidrug resistance–Associated protein involved in anthocyanin transport in *Zea mays*. Plant Cell.

[60] Francisco R.M., Regalado A., Ageorges A., Burla B.J., Bassin B., Eisenach C., Zarrouk O., Vialet S., Marlin T., Chaves M.M. (2013). ABCC1, an ATP binding cassette protein from grape berry, transports anthocyanidin 3-O-glucosides. Plant Cell.

[61] Kang J., Park J., Choi H., Burla B., Kretzschmar T., Lee Y., Martinoia E. (2011). Plant ABC transporters. Arab. Book.

[62] Mentewab A., Stewart C.N. (2005). Overexpression of an *Arabidopsis thaliana* ABC transporter confers kanamycin resistance to transgenic plants. Nat. Biotechnol..

[63] Borghi L., Kang J., Ko D., Lee Y., Martinoia E. (2015). The role of ABCG-type ABC transporters in phytohormone transport. Biochem. Soc. Trans..

[64] Geisler M., Aryal B., Di Donato M., Hao P. (2017). A Critical view on ABC transporters and their interacting partners in auxin transport. Plant Cell Physiol..

[65] Chen P., Li Y., Zhao L., Hou Z., Yan M., Hu B., Liu Y., Azam S.M., Zhang Z., Rahman Z.U. (2017). Genome-wide identification and expression profiling of ATP-binding cassette (ABC) transporter gene family in pineapple (*Ananas comosus*) reveal the role of AcABCG38 in pollen development. Front. Plant Sci..

[66] Xu J.M., Fan W., Jin J.F., Lou H.Q., Chen W.W., Yang J.L., Zheng S.J. (2017). Transcriptome analysis of Al-induced genes in buckwheat (*Fagopyrum esculentum Moench*) root apex: New insight into Al toxicity and resistance mechanisms in an Al ac-cumulating species. Front Plant Sci..

[67] Agrahari R.K., Kobayashi Y., Borgohain P., Panda S.K., Koyama H. (2020). Aluminum-specific upregulation of GmALS3 in the shoots of soybeans: A potential biomarker for managing soybean production in acidic soil regions. Agronomy.

[68] Bernard D.G., Cheng Y., Zhao Y., Balk J. (2009). An allelic mutant series of ATM3 reveals its key role in the biogenesis of cytosolic iron-sulfur proteins in *Arabidopsis*. Plant Physiol..

[69] Kawahara Y., de la Bastide M., Hamilton J.P., Kanamori H., McCombie W.R., Ouyang S., Schwartz D.C., Tanaka T., Wu J., Zhou S. (2013). Improvement of the *Oryza sativa Nipponbare* reference genome using next generation sequence and optical map data. Rice.

[70] UniProt Consortium (2015). UniProt: A hub for protein information. Nucleic Acids Res..

[71] Eddy S.R. (2011). Accelerated profile HMM searches. PLoS Comput. Biol..

[72] Finn R.D., Clements J., Eddy S.R. (2011). HMMER web server: Interactive sequence similarity searching. Nucleic Acids Res..

[73] Elisabeth G., Alexandre G., Christine H., Ivan I., Appel R.D., Amos B. (2003). Expasy: The proteomics server for in-depth protein knowledge and analysis. Nucleic Acids Res..

[74] Hu B., Jin J., Guo A.-Y., Zhang H., Luo J., Gao G. (2015). GSDS 2.0: An upgraded gene feature visualization server. Bioinformatics.

[75] Chen C., Chen H., Zhang Y., Thomas H.R., Frank M.H., He Y., Xia R. (2020). TBtools: An integrative toolkit developed for in-teractive analyses of big biological data. Mol. Plant.

[76] Bailey T.L., Elkan C. (1995). The value of prior knowledge in discovering motifs with MEME. Proc. Int. Conf. Intell. Syst. Mol. Biol..

[77] De Castro E., Sigrist C.J., Gattiker A., Bulliard V., Langendijk-Genevaux P.S., Gasteiger E., Bairoch A., Hulo N. (2006). ScanProsite: Detection of PROSITE signature matches and ProRule-associated functional and structural residues in proteins. Nucleic Acids Res..

[78] He Z., Zhang H., Gao S., Lercher M.J., Chen W.-H., Hu S. (2016). Evolview v2: An online visualization and management tool for customized and annotated phylogenetic trees. Nucleic Acids Res..

[79] Blanc G., Wolfe K.H. (2004). Widespread paleopolyploidy in model plant species inferred from age distributions of duplicate genes. Plant Cell.

[80] Li Z., Jiang H.Y., Zhou L.Y., Deng L., Lin Y.X., Peng X.J., Yan H., Cheng B. (2014). Molecular evolution of the HD-ZIP I gene family in legume genomes. Gene.

[81] Wang Y., Wang Q., Zhao Y., Han G., Zhu S. (2015). Systematic analysis of maize class III peroxidase gene family reveals a con-served subfamily involved in abiotic stress response. Gene.

[82] Machado F.B., Moharana K.C., Almeida-Silva F., Gazara R., Pedrosa-Silva F., Coelho F.S., Grativol C., Venancio T.M. (2020). Systematic analysis of 1298 RNA-Seq samples and construction of a comprehensive soybean (*Glycine max*) expression atlas. Plant J..

