# Supplementary figures and images for: Genome-Wide Identification of Soybean ABC Transporters Relate to Aluminum Toxicity
Source: Int J Mol Sci. 2021 Jun 18;22(12):6556. doi: 10.3390/ijms22126556 (PMC8234336; doi:10.3390/ijms22126556)

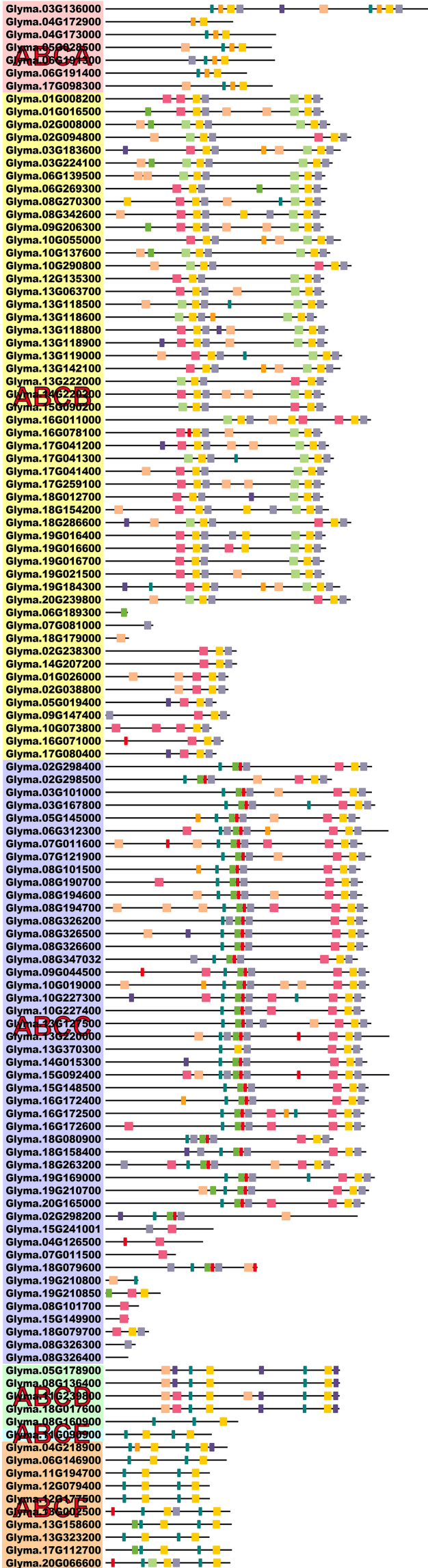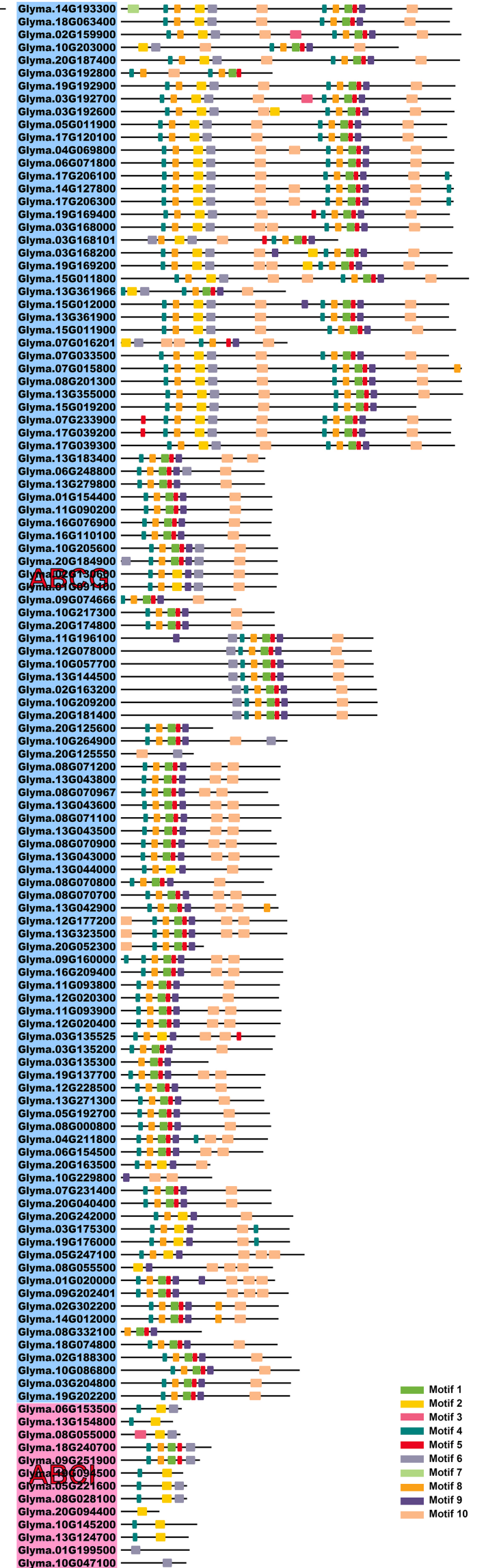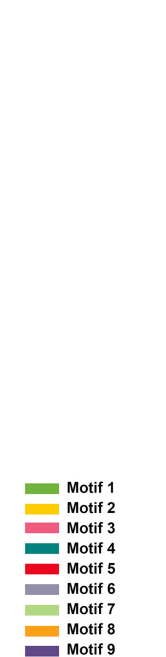

Supplement: Supplementary file 1 [file ijms-22-06556-s001.zip › ijms-1239266-supplementary/Additional file 3 Figure S1.pdf]
